# The Editor's Letter-Box

**Published:** 1920-11-06

**Authors:** 


					To the Editor of Thi; Hospital.
" Is there 110 way for men to be, bat women
Must be half-workers ? " . . .
" . . . I'll write against them,
Detest them, curse them : yet 'tis greater skill,
In a true hate, to pray they have their will :
The very devils cannot plague them better."
?Cymbeline, Act II., Sc. V.
'Sir,?Many vital questions are raised in the article-
on " The Position of Women at Cambridge," published
in your last week's issue, but as your correspondent con-
siders most of these as " subsidiary," it would perhaps,
be well to deal only with the problem upon which she
lays most stress?namely, " whether sex distinction in
education is desirable."
While we hesitate to question the ability of woman to-
attain academical distinctions, and though we do not
' wish to hark back to the smug hypocrisy which dis-
graced the domestic life of the Victorian era, yet we-
cannot ignore the biological destiny of woman. The pic-
ture of women in the Senate House reminds us of Dr.
Johnson's parallel concerning women in the pulpit and
performing dogs?" the marvel is not that they perform
so well, but that they do it at all."
At Cambridge generations of men have evolved a-
130  THE HOSPITAL. November 6, 1920.
Tbe Editor's Letter Box?(continued).
training peculiarly suited to men. Nothing can ever make
women function as men, and if they are to receive the
best education it must be one specific to their own needs.
For women to seek to follow in the way expressly
developed by men, for men, is to impose upon themselves
a training which is of necessity not the best.
Under Scheme "B" women have an opportunity of
organising their own university so that even if their
imagination fails to formulate a new scheme the older
university could be faithfully copied, and both the new
and the old could work unhampered by the " subsidiary "
problems. We are amazed, therefore, to hear that past-
Cambridge women students are strongly opposed to the
idea of a separate university. " Put me amongst the
men " may answer admirably as a music-hall refrain, but
it is not a worthy battle-cry for emancipated woman-
kind.
The argument that co-operation in university life makes
for co-operation later in public life looks very plausible
on paper. So far the only signs of co-operation strikingly
evident are to be found on the floors of the C.U. Dance
Club, the Vingt et Un Club, and on the backwaters of
the Cam. We are a trifle sceptical as to the value of this
as a preparation for public life.?-Yours faithfully,
Cambridge.
At Cambridge Now.

				

